# Mapping Non-Monetary Values in Data Sharing: Development and Validation of an SSH Framework Across Sectors

**DOI:** 10.12688/openreseurope.23548.1

**Published:** 2026-05-26

**Authors:** Margit Hofer, Daniela Fuchs, Eduardo Vyhmeister, Agnieszka Rausch

**Affiliations:** 1 Technology and Knowledge, Centre of Social Innovation, Vienna, Vienna, 1150, Austria; 2 Insight Centre for Data Analytics, University College Cork, Cork, County Cork, Western Gateway Building, Ireland; 3 Supercomputing and Networking Centre, Poznan Supercomputing and Networking Center, 10-Poznań, Greater Poland Voivodeship, 61-139, Poland

**Keywords:** Data Sharing, Social Sciences and Humanities (SSH), Data Governance, EU Data Policy, Non-Monetary Values, European Data Spaces

## Abstract

**Background:**

Data sharing is increasingly recognised as essential for innovation, sustainability, and resilience in Europe. However, existing frameworks primarily focus on technical and economic aspects, neglecting non-monetary values such as trust, ethics, cultural readiness, and organisational capacity. This study addresses this gap through the development and validation of a Social Sciences and Humanities (SSH) Framework for Data Sharing.

**Methods:**

A foresight-oriented, multi-method design combined a systematic literature review, interviews across six European pilots, and participatory foresight activities including visioning, backcasting, and feasibility assessment.

**Results:**

The analysis identified eleven SSH dimensions shaping data sharing: societal, ecological, security, scientific–research, macro-economic, customer–business, policy, ethics, cultural, organisational, and regulatory. Ethics, policy alignment, and scientific–research dimensions showed the highest feasibility across sectors, while cultural and societal dimensions consistently presented the greatest implementation challenges.

**Conclusions:**

The SSH Framework offers an empirically grounded tool for designing inclusive and human-centric data governance. The results indicate strong alignment with EU data policy frameworks while revealing persistent sociocultural barriers. The Framework supports policy design by identifying priority areas for regulation, governance development, and capacity building at the EU, sectoral, and organisational levels, thereby contributing to the EU ambition of trustworthy and socially sustainable data spaces.

## Plain Language summary

Sharing data between organisations and sectors can create many benefits, from developing better services to supporting sustainability and improving public decision-making. But technical solutions and financial incentives are only part of the picture. Societal factors such as trust, ethics, culture, and organisational capacity also play a major role in whether organisations benefit from data sharing.

This study introduces a Social Sciences and Humanities (SSH) Framework for data sharing that highlights these often-overlooked aspects. The framework is based on a review of recent research and was tested in six real-world pilot projects in the fields of energy, telecommunications, agriculture, infrastructure, and meteorology. It brings together 11 key dimensions that influence how data sharing works across different sectors.

Through interviews, workshops, and future-vision exercises, we explored which dimensions matter most in each sector and how feasible they are to implement. Across all pilots, trust, ethics, and strong organisational structures emerged as the most important and actionable elements. Cultural readiness and public engagement were also recognised as important but more difficult to achieve.

The SSH Framework provides practical insights for companies, policymakers, and researchers who want to design data-sharing systems that are inclusive, ethical, and resilient. By considering social and organisational factors alongside technical ones, data sharing can deliver benefits not only for the economy but for society as a whole.

## 1. Introduction: The need for an SSH framework in data sharing

Data sharing is widely recognised as a driver of digital transformation and societal value across sectors including agriculture, energy, telecommunications, infrastructure, and meteorology. European policy initiatives, such as the European Strategy for Data and the Data Governance Act, aim to create interoperable and trustworthy data ecosystems. ^1, 2^ However, implementation remains uneven, as dominant approaches still emphasise technical and economic factors while underestimating the social and institutional conditions that determine whether data sharing is adopted in practice. ^3, 4^

SSH research shows that data sharing is a socio-technical practice shaped by organisational structures, institutional norms, and cultural expectations. ^5, 6^ Technical systems embed social choices that influence perceptions of fairness, accountability, and legitimacy. ^7^ When these non-monetary factors are overlooked, data infrastructures is in risk becoming socially fragile, limiting adoption and trust. ^8^

Recent studies highlight that non-monetary values such as inclusion, ethical responsibility, ecological sustainability, and cultural acceptance are essential for effective data sharing. ^9– 11^ However, most analyses address these aspects in isolation or within single sectors, limiting cross-sector learning and transferability of governance insights. ^12, 13^ As Europe moves toward federated data spaces, it becomes clear that technical interoperability alone cannot resolve challenges related to legitimacy, trust, and power asymmetries. ^3, 14^

To address this gap, this study develops and validates a Social Sciences and Humanities (SSH) Framework for Data Sharing. The framework integrates insights from a systematic literature review, stakeholder interviews across six European pilot sectors, and participatory foresight activities. Foresight provides a structured approach for exploring future governance needs and implementation challenges, ^15, 16^ aligning with the EU’s commitment to anticipatory governance. ^17^

1. RQ1: What SSH dimensions significantly influence data-sharing practices in the context of European data policy?
2. RQ2: Does the SSH Framework demonstrate applicability for generating sector-specific visions of trusted, value-generating data sharing aligned with EU regulatory goals?
3. RQ3: Which SSH dimensions pose greater implementation challenges, according to stakeholders’ feasibility assessments of the corresponding recommendations?

This study contributes to the development of human-centric, socially legitimate, and resilient data ecosystems by:

1. Developing the SSH Framework, with eleven non-monetary dimensions rooted in literature;
2. Empirically validating the framework across six diverse pilots in five thematical domains;
3. Applying participatory foresight methods to generate sector-specific visions and assess the feasibility of related recommendations.

Through cross-sector validation and feasibility assessment, this study contributes an operational governance framework for understanding non-monetary drivers of data sharing. The SSH Framework complements EU data policy by identifying where regulatory alignment is strong and where sociocultural capacity building is necessary to achieve inclusive and resilient European data ecosystems.

## 2. Theoretical background: Situating non-monetary dimensions in data sharing

### 2.1 Data sharing as a socio-technical practice

Conventional approaches to data sharing primarily focus on technical interoperability, data quality, and economic value creation. ^4, 18^ However, these factors alone fail to explain differences in adoption and sustainability across data-sharing initiatives. ^19^ SSH research conceptualises data sharing as a socio-technical practice shaped by organisational norms, institutional arrangements, and trust relations. ^3, 6, 20^ Technical systems embed social choices that influence perceptions of legitimacy, accountability, and fairness. ^7^ Without attention to these relational dimensions, data infrastructures risk becoming socially fragile even when technically functional. ^21^

### 2.2 Non-monetary value and gaps in existing data-sharing frameworks

Much of the data-sharing literature continues to prioritise technical interoperability and economic value creation. ^4, 18^ While these dimensions remain important, they are insufficient to explain variation in adoption, sustainability, and governance effectiveness across data ecosystems.

A growing body of research identifies non-monetary values as central to data sharing, including trust, ^22^ ethical responsibility, transparency, ^23^ social inclusion, and environmental sustainability. ^9, 10, 24^ These values influence not only user behaviour but also institutional legitimacy and public acceptance. From an SSH perspective, data infrastructures are not neutral artefacts but socio-technical systems shaped by governance arrangements, organisational routines, and cultural norms. ^3, 6, 19^

Despite this recognition, existing frameworks typically address social and ethical dimensions in isolation and within sector-specific contexts. Comparative and transferable governance models remain limited, ^25^ and cross-sector learning is constrained. ^12, 13^ Furthermore, many conceptual approaches remain descriptive and lack operational tools for assessing implementation readiness or institutional feasibility.

This fragmentation is particularly problematic in the European context, where policy initiatives aim to support cross-sectoral interoperability through federated data spaces. ^1^ To strengthen the theoretical foundation of the SSH Framework, the identified dimensions were situated within a comprehensive theoretical context, drawing on socio-technical systems theory ^21^ and governance frameworks that emphasise social legitimacy in digital transformation. ^6, 7^ The SSH dimensions were integrated with concepts from institutional theory, which suggests that data-sharing practices are shaped by the interplay of technical, social, and organisational factors. ^3^ Moreover, the theoretical grounding will expand by referencing established models of data governance, such as the Governance of Open Data, ^14, 25^ and incorporate perspectives from literature on trust and transparency in digital ecosystems. ^9, 10^ This will provide a clearer theoretical justification for the inclusion of the SSH dimensions, illustrating how they build on and extend existing scholarship while contributing to the emerging field of non-monetary data governance.

However, technical federation alone cannot resolve legitimacy deficits, power asymmetries, or trust issues. ^3, 14^ Without addressing these conditions, data ecosystems risk becoming technically advanced but socially unstable.

In addition, the concept of value in data sharing is often narrowly framed in economic terms. This overlooks how data practices affect societal well-being, organisational capacity, and governance resilience. ^10, 11, 24^ Ethical governance, cultural readiness, and institutional trust are therefore not secondary considerations but constitutive elements of sustainable data infrastructures.

The lack of integrated SSH frameworks capable of linking conceptual insight to applied governance remains a major limitation in existing scholarship. Few approaches offer structured methods to translate social and ethical concerns into operational guidance for policymakers and practitioners.

This study addresses this gap by proposing a framework that integrates eleven non-monetary SSH dimensions into a single analytical model. By combining conceptual grounding with empirical validation and foresight-based application, the framework supports structured comparison across sectors and enables assessment of governance readiness beyond technical feasibility alone.

### 2.3 Fragmented data ecosystems and the need for integrated frameworks

Despite growing recognition of SSH dimensions, most studies examine social and ethical issues within single-sector contexts. As a result, cross-sectoral learning remains limited and governance models are rarely transferable. ^12, 13^

European initiatives aim to replace siloed systems with interoperable and federated data spaces. ^1^ Yet technical federation alone cannot resolve structural inequalities, legitimacy deficits, or power asymmetries. ^3, 14^ This reveals the need for frameworks capable of integrating social, organisational, and regulatory dimensions into cross-sector governance analysis. ^25^

### 2.4 Foresight as a bridge between policy and practice

While most data-sharing studies are retrospective, foresight offers an anticipatory, participatory approach to exploring future risks and designing adaptive strategies. ^15, 16^ The European Commission’s Strategic Foresight Reports ^17^ promote foresight to align digital development with societal priorities. Foresight in data governance enables stakeholders to test frameworks, assess feasibility, and co-develop recommendations reflecting both societal expectations and operational constraints. ^26^

Thus, foresight can act as a bridge between conceptual analysis and practical governance and decision making, ^27^ enabling collaborative testing of frameworks and anticipatory policy design.

## 3. Methodology

### 3.1 Research design

**Figure 1.**
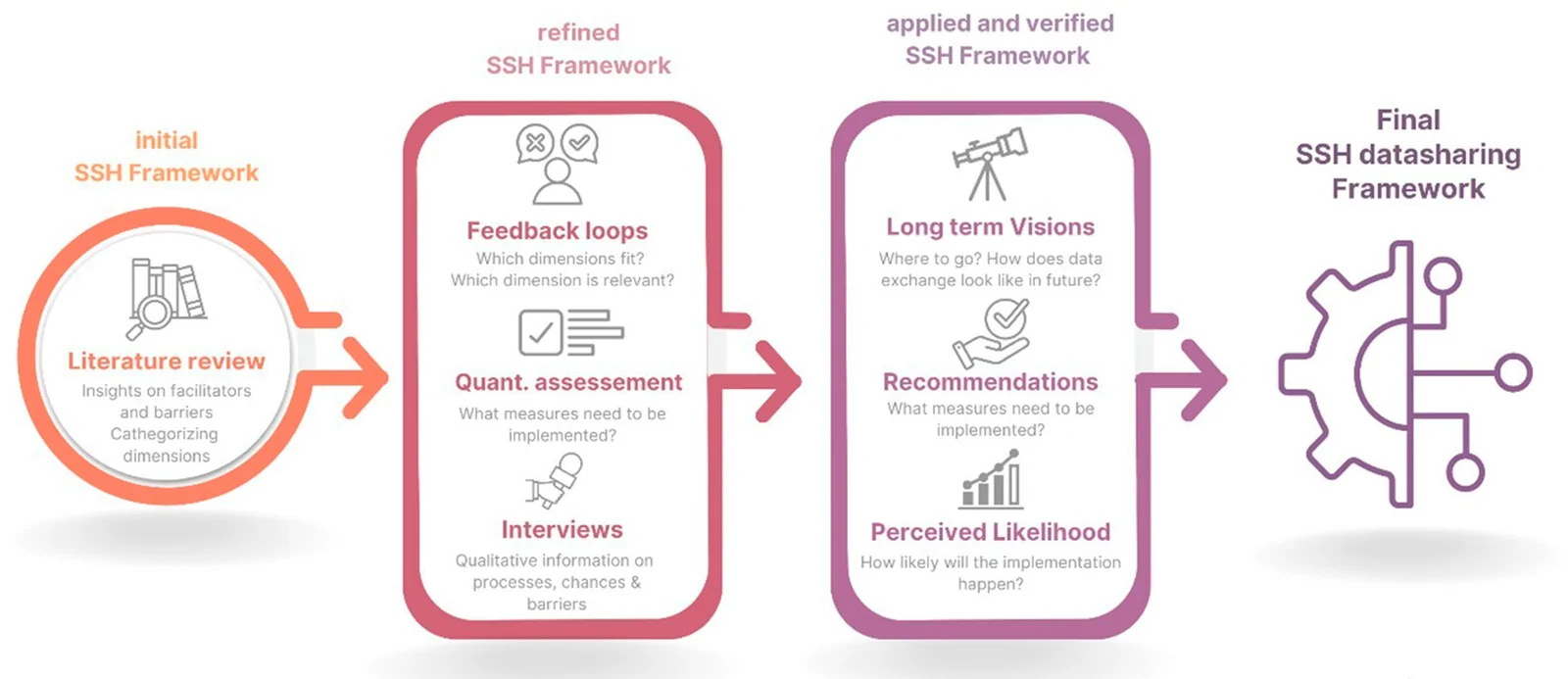
Overview of research methodology developing DATAMITE SSH data sharing framework.

A three-stage sequence created a robust evidence base (see Figure 1 for an overview of the mixed method applied) for understanding non-monetary impacts within data-sharing ecosystems and for deriving actionable recommendations aligned with EU data policy goals.

**(a) Exploration and Conceptual Mapping through a Systematic Literature Review**

A systematic and transparent literature review was conducted as part of the DATAMITE foresight process. Its purpose was to identify non-monetary impacts of data sharing, explore sector-specific patterns, and establish a conceptual foundation for subsequent empirical inquiry.

To enhance transparency, the following steps were followed:

• The search strategy was clearly defined using the PICOC methodology ^28, 29^ (Population, Intervention, Comparison, Outcomes, Context), ensuring the inclusion of studies with rigorous methodological approaches and relevance to the research questions.
• A structured search protocol was applied across Scopus and Google Scholar, covering peer-reviewed literature published between 2014 and 2024.
• A total of 253 records were assessed, with 40 studies selected based on title, abstract, and keyword analysis. This process was documented to ensure reproducibility and provide clarity on the study selection criteria.

The review included both deductive and inductive coding methods, ensuring comprehensive insights into the literature corpus. The coding process was documented to allow for reliable replication of the categorisation of sectors, dimensions, data types, and other variables.

**(b) Empirical Refinement and Sectoral Contextualisation through Stakeholder Interviews and Feedback Loops**

The SSH framework was iteratively refined through feedback loops with pilot stakeholders. Two rounds of feedback were conducted, and an online discussion allowed pilot partners to comment on definitions, applicability, and missing considerations. A total of six semi-structured interviews (one per pilot) were conducted between April and October 2024 with representatives from the six DATAMITE pilot cases. The interview protocol was standardised to ensure consistency and transparency across sectors.

Interviews were recorded, transcribed, and analysed in MAXQDA, using a mixed deductive-inductive coding approach. Predefined categories from the literature guided initial coding, while inductive subcodes captured emergent themes. Coding reliability was ensured through inter-coder agreement, with final categorisation confirmed through consensus among the research team. A total of 327 coded references were generated, and stakeholders were given the opportunity to validate the final analysis.

**(c) Validation and Forward-Looking Assessment through Participatory Foresight**

The third methodological pillar involved strategic foresight activities ^30, 31^ to validate and operationalise the SSH Framework. This participatory process unfolded in three stages:

1. Vision Development: Each pilot team co-created a 5- to 10-year desirable future for trusted and value-generating data sharing in their sector.
2. Backcasting and Recommendation Formulation: The backcasting workshop allowed participants to develop enabling actions, governance structures, and policy measures required to achieve their vision.
3. Feasibility Assessment: Stakeholders rated the perceived feasibility of recommendations using a six-point Likert scale (1 = very low, 6 = very high), which was discussed and agreed upon within their pilot teams. Feasibility scores reflect sector-specific readiness for implementation rather than actual execution.

The results of these foresight activities were analysed and incorporated into the SSH Framework, providing actionable recommendations for future data governance practices.

**Reliability and Reproducibility**

To ensure the reliability and reproducibility of the findings, all coding procedures (literature review, interviews, and feasibility assessments) were conducted according to clearly defined protocols. Feasibility scores and recommendations were based on clear, documented criteria and involved multiple rounds of feedback to reduce bias and ensure that findings reflected diverse stakeholder perspectives.

**Study Context: DATAMITE pilots**

DATAMITE, funded under Horizon Europe (Grant No. 101092989), develops modular and interoperable solutions for data exchange, governance, and quality. The project includes six pilots in five thematical domains: telecommunications (pilot OTE), energy (pilot HEDNO and pilot E-REDES), agriculture (pilot WODR, PSNC), infrastructure (pilot GIDI), and meteorology (pilot CINECA).

These pilots provide the empirical foundation for this study by enabling cross-sector comparison of both technical and non-monetary aspects of data sharing. They represent diverse institutional settings and governance maturities, allowing systematic analysis of how social, organisational, and ethical factors affect feasibility and readiness across sectors. Based on these six pilots, the SSH Framework was iteratively refined and validated using evidence generated within these pilot contexts.

### 3.2 Data collection

**Literature Review for initial SSH Framework**

A comprehensive systematic literature review was conducted as part of the DATAMITE foresight process. Its aim was to identify non-monetary impacts of data sharing, explore sector-specific patterns, and establish a conceptual foundation for subsequent empirical inquiry.

To construct the queries, the PICOC methodology – Population, Intervention, Comparison, Outcomes, and Context was applied. The PICOC methodology comprises five parameters for defining search terms:

• Population: the specific group of interest, i.e., manuscripts from which the research question is formulated, here SSH related manuscripts;

• Intervention: the approach applied in the study, like defining processes of data sharing to be included in the search;

• Comparison: the differentiation of the topic at stake, i.e. in terms of benefits and risks;

• Outcome (not to be defined since no specific outcomes are considered);

• Contexts: the social contexts in which benefits and risks occur, thus, limiting the search to specific sectors and dimensions.

The review followed structured search protocols across Scopus and Google Scholar, covering peer-reviewed literature published between 2014 and 2024. Search terms included non-monetary, open data or data sharing, as well as different dimensions, and sectors considered in the search (see Table 1 for the search string per database).

**Table 1.** Search string per database.

Database	Records	Search string
Google Scholar	112	allintitle: (“macroeconomic” OR “Environmental” OR “Scientific” OR “Societal” OR “Security” OR “Risk”)(“Non-Monetary” OR “Open Data” OR “Data Sharing”)(“Data”)(“Urban” OR “Manufacturing” OR “Retail” OR “Media” OR “Entertainment” OR “Finance” OR “Banking” OR “Energy” OR “Weather” OR “Agriculture” OR “Telecommunication” OR “Cultural” OR “Health” OR “Medicine” OR “Government” OR “Retail” OR “Transport” OR “Insurance” OR “Public Services”)
Scopus	33	TITLE ((“macroeconomic” OR “Environmental” OR “Scientific” OR “Societal” OR “Security” OR “Risk”) AND (“Non-Monetary” OR “Open Data” OR “Data Sharing”) AND data AND (“Urban” OR “Manufacturing” OR “Retail” OR “Media” OR “Entertainment” OR “Finance” OR “Banking” OR “Energy” OR “Weather” OR “Agriculture” OR “Telecommunication” OR “Cultural” OR “Health” OR “Medicine” OR “Government” OR “Retail” OR “Transport” OR “Insurance” OR “Public Services”)) AND PUBYEAR >2013 AND PUBYEAR <2025
	152	TITLE-ABS-KEY ((“macroeconomic” OR “Environmental” OR “Scientific” OR “Societal” OR “Security” OR “Risk”) AND (“Non-Monetary” OR “Open Data” OR “Data Sharing”) AND data AND (“Urban” OR “Manufacturing” OR “Retail” OR “Media” OR “Entertainment” OR “Finance” OR “Banking” OR “Energy” OR “Weather” OR “Agriculture” OR “Telecommunication” OR “Cultural” OR “Health” OR “Medicine” OR “Government” OR “Retail” OR “Transport” OR “Insurance” OR “Public Services”)) AND PUBYEAR >2013 AND PUBYEAR <2025 AND (EXCLUDE (SUBJAREA, “COMP”) OR EXCLUDE (SUBJAREA, “CENG”) OR LIMIT-TO (SUBJAREA, “SOCI”)) AND (LIMIT-TO (EXACTKEYWORD, “Data Sharing”) OR LIMIT-TO (EXACTKEYWORD, “International Cooperation”) OR LIMIT-TO (EXACTKEYWORD, “Open Science”) OR LIMIT-TO (EXACTKEYWORD, “Data Management”) OR LIMIT-TO (EXACTKEYWORD, “Information Management”) OR LIMIT-TO (EXACTKEYWORD, “Access To Information”))

The initial number of records (n = 297) was reduced to a pool of 253 records by reducing doubles, restricting the search to studies in English. Out of these 253, 40 studies were selected by a core team based on title, abstract, and keywords, using criteria of methodological rigor and relevance to the research question. The resulting 40 papers were assigned to different categories, such as dimensions, sectors, and where applicable nature of data, process and modes of sharing data and target groups and users of articles (with multiple assignments possible).

Subsequently, these studies were analysed quantitatively and qualitatively, combining deductive and inductive approaches for comprehensive insights into the literature corpus. The final coding system comprised: sectors (where data was shared), dimensions of data sharing, and target groups, data users, modes of data sharing, types of data to be shared (if specified). Moreover, it covered associated benefits, risks & challenges (which were further inductively differentiated), resource allocation, and additional codes (i.e., interesting aspects that occurred during the inductive analysis). Most codes proved consistent among literature, except for specificities regarding sectors or data (e.g., Open Government Data or sharing genomic data raised specific challenges).

Using thematic analysis, ^32^ an initial set of six benefit dimensions was expanded to eleven: societal, ecological, security, scientific–research, macro-economic, customer–business, policy, ethics, cultural, organisational, regulatory effects. This expansion served as the conceptual backbone for the empirical phases that followed and the subsequent plot analysis indicated the co-occurrence of topics, in particular among dimensions, sectors and impacts (i.e., specific benefits and risks).

The review revealed cross-sectoral patterns and gaps. Urban/public services, medicine/health, and science/research were most frequently addressed, reflecting established data-sharing cultures and openly accessible data. Other sectors central to DATAMITE, such as energy and agriculture, were less represented, underscoring the need for empirical validation within these domains.

Although the study is based on six diverse pilot sectors, including energy, agriculture, telecommunications, infrastructure, and meteorology, we acknowledge that sectors like health and manufacturing were not as well represented. To address this, future research will apply the SSH Framework in additional sectors, particularly in healthcare and manufacturing, to test its cross-sector applicability and scalability. Initial consultations with stakeholders in these sectors suggest that the framework’s dimensions of ethics, trust, and organisational readiness are likely to resonate similarly across other domains. Additionally, the framework will be iteratively refined in these new contexts to ensure its flexibility and applicability beyond the pilot sectors. This broader application will strengthen the framework’s relevance and highlight whether sector-specific adjustments are necessary or if the SSH dimensions remain universally applicable.

**Feedback loops, Stakeholder Interviews and Estimated Impact for Iterative Refinement of SSH Framework**

Building on the literature review, the SSH Framework was iteratively refined through feedback loops with pilot stakeholders. Two dedicated feedback rounds and an additional online discussion enabled pilot partners to comment on definitions, applicability, and missing considerations. Continuous input throughout the project ensured that practical insights and sector-specific challenges informed the framework.

To contextualise and deepen the conceptual findings, six semi-structured interviews were conducted between April and October 2024 with representatives from the six DATAMITE pilot cases. Considering the breadth of scope within the pilots, as the DATAMITE pilot cases are focused on technical aspects rather than thematic coherence, we decided to focus on the project’s range due to the internal variability of the DATAMITE case studies. Participants included project leads, data managers, and policy or public-sector partners from all pilot domains. The interviews lasted approximately one hour and were held online.

The interview protocol explored:

• relevance of the identified SSH dimensions within each sector,
• barriers and enablers to data sharing,
• examples of institutional or cultural resistance,
• and perceived long-term value.

The interviews were recorded, transcribed, and analysed in MAXQDA using a mixed deductive–inductive coding approach: predefined categories from the literature guided initial coding, while inductive subcodes captured emergent themes. A total of 327 coded references were generated across the eleven dimensions and interview partners had the chance to provide feedback on the final analysis.

To complement and validate qualitative insights, a structured questionnaire was administered to each pilot stakeholder representative. Participants from the pilots rated the expected benefits of data sharing in their sector on a 0–6 Likert scale, where 0 indicates no expected benefit and 6 indicates very high benefit. The results were visualised in a radar chart to facilitate cross-sector comparison; where more than one rating was submitted (pilots energy and agriculture), the mean was considered.

**Applying and Validating SSH framework by Participatory Foresight**

The third methodological pillar applied strategic foresight to validate and operationalise the SSH Framework. This participatory process unfolded in three main stages:

1. **Vision Development:**
  Each pilot team co-created a 5 to 10 year “desirable future vision” for trusted and value-generating data sharing in their sector. Stakeholders were guided through structured questions during the interviews and in written format. These visions were again validated in a face-to-face backcasting workshop with 46 partners of DATAMITE. The material was analysed and mapped for comparison.
2. **Backcasting and Recommendation Formulation:**
  The backcasting workshop involved participants from all six pilots and data sharing experts of DATAMITE. Based on the validated vision, participants identified enabling actions, governance structures, and policy measures required to reach their visions. Based on these needed actions, participants collectively formulated 49 recommendations, including 10 cross-dimensional recommendations.
3. **Feasibility Assessment:**
  Finally, each pilot self-assessed the feasibility (i.e., *perceived* ability of implementation, *not actual* implementation) of its recommendations using a six-point Likert scale (1 = very low, 6 = very high). Stakeholders discussed each recommendation within their pilots and jointly agreed on a rating. Feasibility scores indicate each sector’s perceived readiness and structural constraints rather than confirming that implementation will occur.

These foresight activities enabled systematic cross-sector comparison and translated conceptual dimensions into actionable steps.

### 3.3 Data analysis and integration

The methodological combination yielded a robust foundation for understanding and operationalising non-monetary dimensions of data sharing. Triangulation across all components ensured internal consistency despite the limited number of use cases.

• The literature review identified conceptual dimensions.
• The interviews, feedback loops, and questionnaire refined their empirical relevance and contextual expression.
• The foresight workshops validated practical applicability and yielded sector-specific recommendations and feasibility assessments.

Data integration was performed through a matrix-based analysis, aligning sectoral findings along the eleven SSH dimensions. The approach enabled identification of cross-cutting patterns, divergences, and implementation barriers. The integrative design enabled the SSH Framework to evolve from a conceptual typology into a validated, multi-dimensional tool for analysing and guiding data-sharing practices across diverse domains.

## 4. Results

This section presents the empirical results of the three methodological pillars: (1) literature review, (2) stakeholder interviews and questionnaire, and (3) participatory foresight incl. Recommendations. Findings are structured along the three research questions.

### 4.1 Identification and cross-sectoral validation of SSH dimensions (RQ1)

The multi-method analysis confirmed findings from Scholl, 2021, ^6^ Zuiderwijk et al. 2017 ^20^ and Micheli et al. 2020, ^33^ that data sharing is shaped by technical, economic, and social factors. Addressing RQ1, the triangulation of literature, interviews, and pilot evidence resulted in eleven SSH dimensions that collectively characterise socially sustainable data-sharing ecosystems.

**4.1.1 Exploration and conceptual mapping of non-monetary dimensions**

The thematic synthesis of 40 peer-reviewed studies yielded an initial set of eight non-monetary dimensions, later expanded to eleven: societal, ecological, security, scientific–research, macro-economic, customer–business, policy, ethics, cultural, organisational, and regulatory. These dimensions represent a range of value dimensions associated with data sharing beyond financial return, including fairness, inclusion, trust, accountability, and knowledge production.

The literature included evidence from telecommunication, energy, urban/public services, meteorology, and agriculture, supplemented by healthcare, manufacturing, finance, media, and cultural heritage. Urban/public services, healthcare, and scientific–research contexts were most frequently reported. Energy and agriculture were less represented in the literature corpus.

**4.1.2 Empirical refinement and sectoral illustrations (Interviews, feedback loops and survey)**

The eleven dimensions of the SSH framework were examined across the DATAMITE pilots, confirming their relevance and interconnections in practice. Sectoral illustrations highlight how non-monetary factors shape data practices. In agriculture, stakeholders expressed reluctance to share data due to concerns over ownership, control, and reuse arrangements (Societal), while meteorological services leveraged data infrastructures for early-warning systems and climate analysis (Ecological). Energy, telecommunications, and infrastructure pilots applied integrated security architectures to manage risk and access (Security), and energy-sector applications focused on predictive maintenance and system optimisation (Scientific–research). Agricultural stakeholders explored data-driven market mechanisms and service-based revenue models (Macro-economic), and telecommunications pilots used data analytics for customer insights and process automation (Customer–business). Energy and meteorology cases highlighted interactions between data infrastructures and sectoral planning processes (Policy), and pilots reported governance arrangements addressing accountability, fairness, and responsible data use (Ethics). Organisational initiatives promoted data literacy and supportive cultural practices (Cultural), with formal roles for data governance established in infrastructure and energy pilots (Organisational). All pilots aligned with European data regulations and sector-specific legal frameworks (Regulatory). These empirical examples demonstrate how the eleven dimensions operate in real-world settings, revealing the synergies and tensions that influence adoption, trust, and effective use of data infrastructures. Taken together, the findings confirm that data sharing is a socio-technical practice shaped not only by technical systems but also by organisational structures, institutional norms, and cultural expectations, highlighting the importance of integrating non-monetary factors into the design and governance of data infrastructures. ^5– 8^

**4.1.2.1 Interdependencies among SSH dimensions**

The SSH dimensions influence and reinforce one another.

• Governance/trust: Clear, transparent governance builds trust; opaque or fragmented structures erode it and reduce willingness to share.
• Privacy/ethics: Privacy protection is a core ethical concern, particularly in sectors like healthcare and agriculture. Balancing openness with privacy requires ethical governance that respects regulatory and societal norms.
• Societal/cultural: Societal aims of equity and fairness are mediated by cultural norms. In agriculture, cultural resistance to transparency can block fair benefit distribution unless explicitly addressed.
• Macro-economic/customer–business: New markets and innovation ecosystems often stem from improved service quality and competitiveness, creating feedback loops where customer-level gains support broader economic impacts.
• Organisational/regulatory: Organisational readiness depends on regulatory clarity. As organisations adopt data-sharing practices, they must align processes with frameworks such as the Data Governance Act and the European Strategy for Data, while regulations must remain flexible enough to accommodate sectoral diversity.

**Figure 2.**
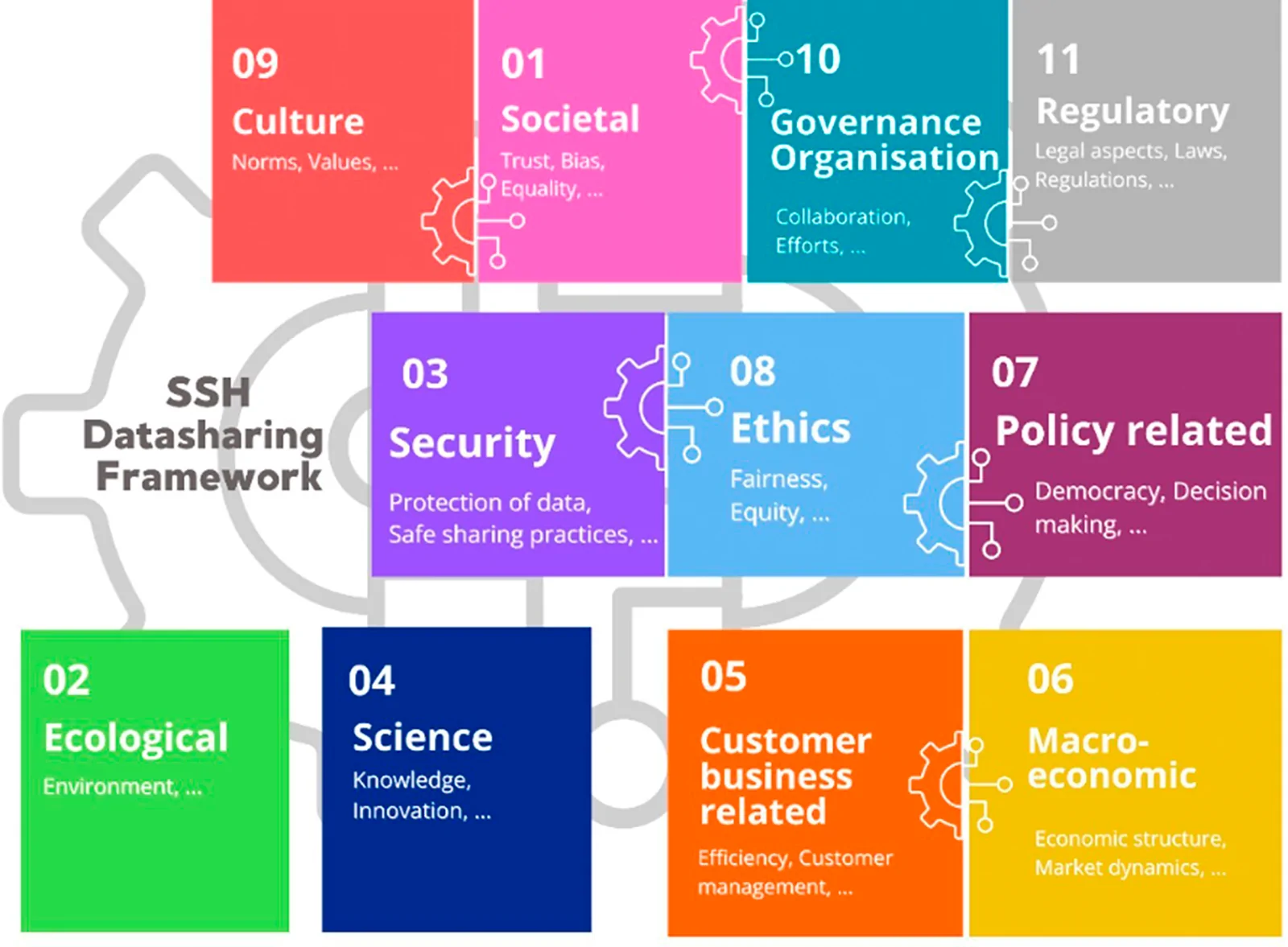
SSH framework for data sharing with 11 dimensions reflecting interlinkages.

Figure 2 presents the interdependencies across dimensions as identified in the pilot contexts.

**4.1.2.2 Cross-sectoral validation and perceived relevance (Interviews & Survey)**

The interviews and survey responses validated that all dimensions were reported as relevant across sectors.

Sectoral profiles included:

• Energy: Ethical governance and organisational arrangements for regulatory compliance.
• Telecommunications: Organisational change initiatives related to system integration and data governance.
• Agriculture: Governance arrangements addressing licensing, ownership, and institutional fragmentation.
• Infrastructure: Security systems linked with operational processes and organisational roles.
• Meteorology: Data ingestion and modelling infrastructures supporting scientific–research dimensions.

**Figure 3.**
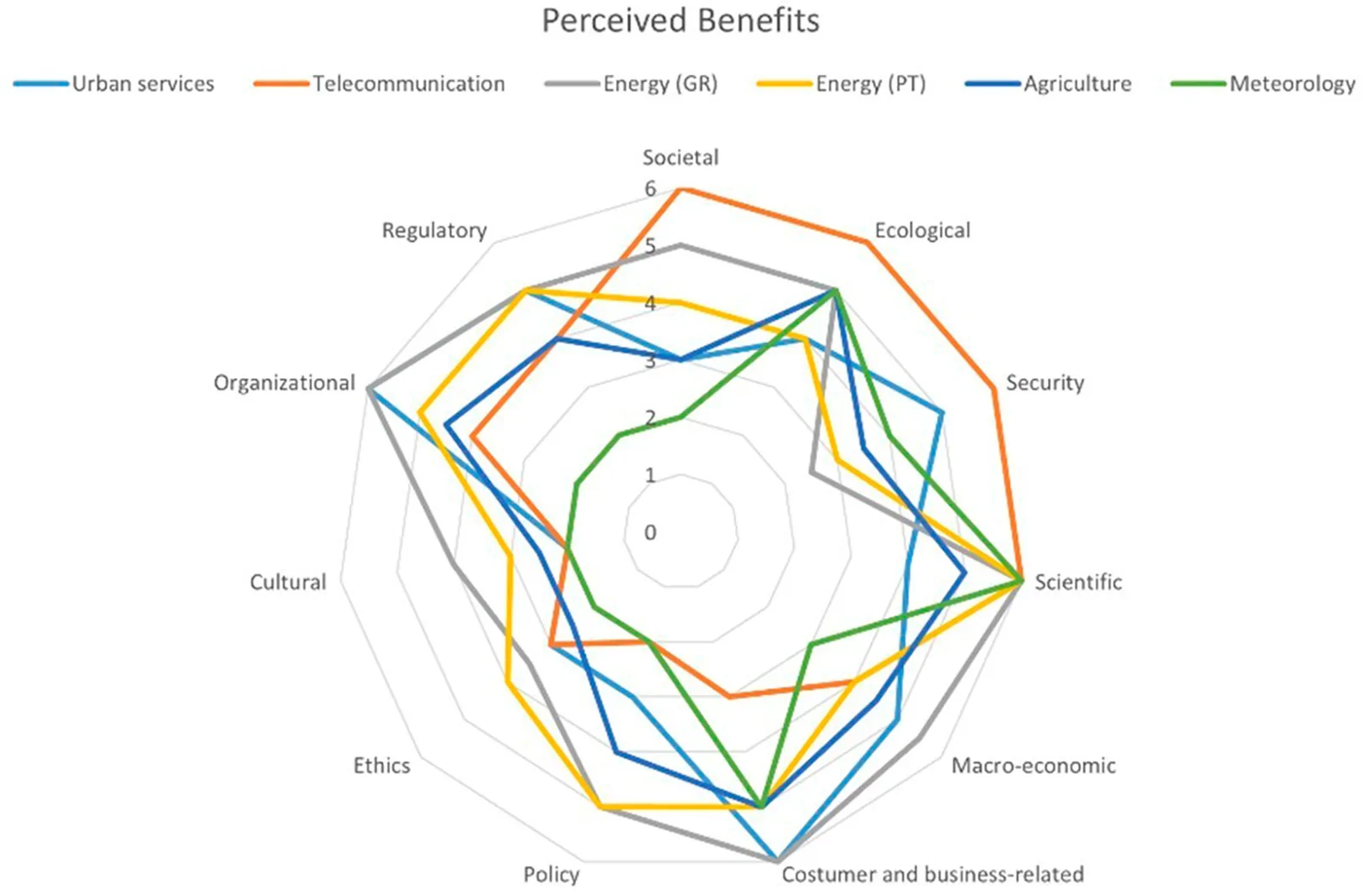
Perceived benefits for each dimension per pilot sector.

The structured questionnaire asked participants to rate the expected relevance of SSH dimensions on a scale from 0 (no relevance) to 6 (very high relevance). Figure 3 visualises dimension ratings for each pilot.

No dimension received a relevance score of zero across cases. Ratings varied by sector, indicating differing distributions of perceived relevance across dimensions.

Data are based on questionnaire responses collected as described in Section 3.3.2.

### 4.2 Pilot-specific future visioning (RQ2)

To address RQ2, whether the SSH Framework is applicable for generating sector-specific visions, all pilot teams conducted structured foresight exercises to co-create a desirable 5–10 year future for data sharing in their sector. Each process used the SSH frameworks dimensions as reference points to guide discussion and scenario development.

Across pilots, the SSH Framework helped structure conversations around the 11 dimensions. Visioning outputs varied by sector, reflecting differences in institutional maturity, regional context, and strategic priorities:

• Technical infrastructure and scalability
• Societal and environmental priorities
• Economic development and competitiveness
• Governance maturity and institutional coordination

**4.2.1 Cross-pilot priorities in future visions**

Despite differences across domains, the visions revealed several recurring priorities:

• Infrastructure capacity: Scalable and interoperable systems
• Data quality: Reliable, structured, and accessible datasets
• Governance mechanisms: Transparency, privacy protection, and accountability
• Collaboration: Cross-organisational and cross-sector partnerships
• Service development: Data-based products and public services
• Regulatory alignment: Compliance with European frameworks
• Incentive mechanisms: Business models and benefit-sharing mechanisms
• Organisational capacity: Staff skills and governance roles

### 4.3 Anticipated implementation likelihood of sector-specific recommendations (RQ3)

To address RQ3 (which SSH dimensions pose greater implementation challenges), participants evaluated the *perceived feasibility* of sector-specific recommendations as an indicator of implementation difficulty, not as evidence of execution. This feasibility analysis highlights which dimensions are more readily actionable and which require additional support.

Stakeholders rated the perceived feasibility of recommendations using a six-point Likert scale (1 = very low, 6 = very high). Ratings reflect perceived implementability rather than observed execution.

**Figure 4.**
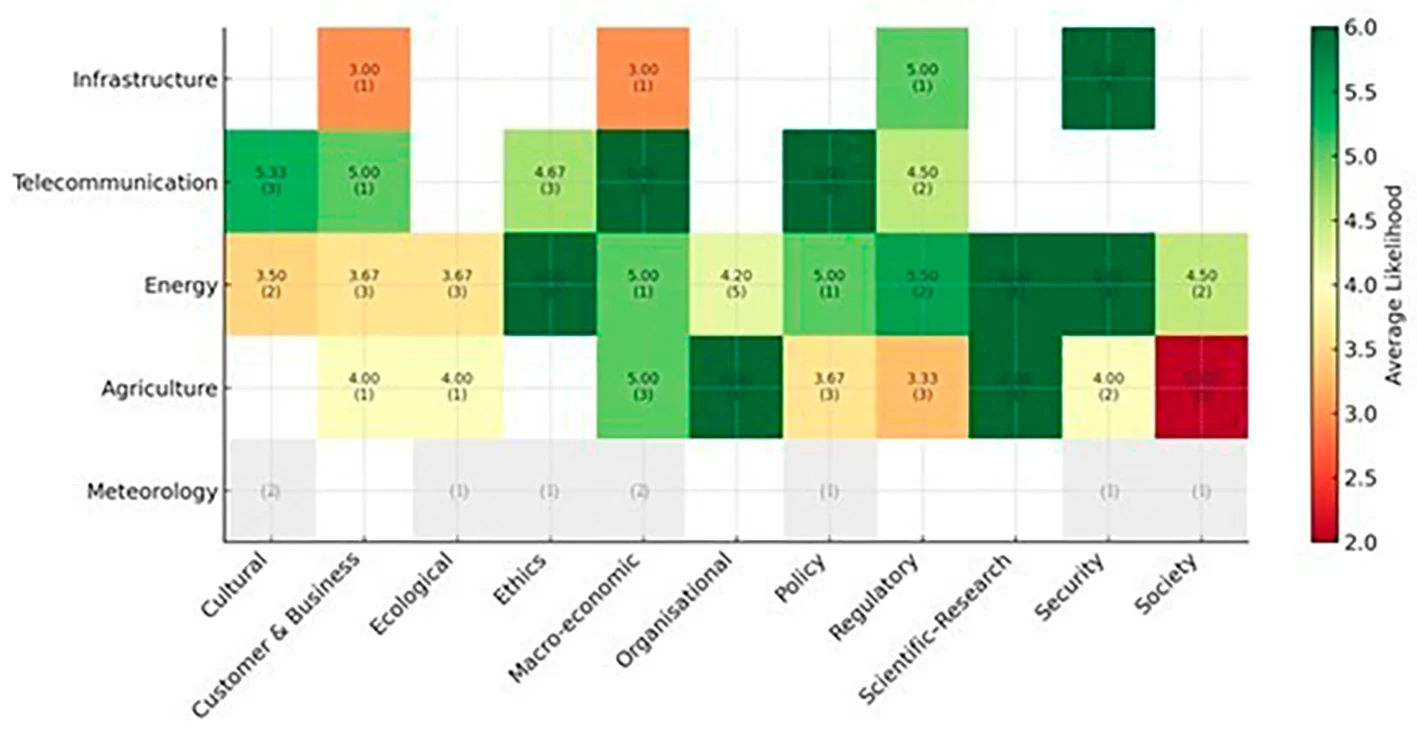
Feasibility of implementing SSH-dimension-related recommendations across six DATAMITE pilots.

Figure 4 summarises the feasibility assessment conducted during the backcasting workshop in each of the six DATAMITE pilots.

Across pilots:

• Ethics, policy, and scientific–research generally exhibited higher feasibility values.
• Societal and cultural dimensions received lower values across multiple sectors.
• Organisational and regulatory feasibility varied by sector.

These scores indicate variation in perceived readiness across dimensions and domains.

## 5. Limitations

While the study offers a comprehensive and empirically grounded SSH Framework for Data Sharing, several limitations should be acknowledged.

The analysis is based on six Horizon Europe DATAMITE pilots, representing well-resourced sectors committed to data sharing. These sectors’ higher levels of institutional readiness and strategic capacity may introduce an engagement bias, limiting the generalizability of findings to less mature or less resourced sectors or non-EU contexts. Sectors with fewer resources or regulatory support may face significantly different barriers.

The foresight-based feasibility assessment relied on self-reported stakeholder ratings, which reflect participants’ perceptions rather than independent verification. While the feasibility ratings were derived from stakeholder perceptions within the pilots, future work will supplement these self-reported assessments by incorporating independent third-party evaluations and post-implementation analyses. This will include follow-up surveys and expert validation, allowing for a more nuanced understanding of the framework’s actual implementation challenges. Additionally, the feasibility scores will be triangulated with real-world metrics, such as adoption rates and operational impact, to verify the accuracy of these ratings. By using such multi-source data, the framework’s potential for real-world implementation can be more robustly evaluated and its feasibility more reliably assessed. These ratings could be influenced by optimism bias or varying interpretations of feasibility across sectors, so they should be considered indicative rather than definitive measures of implementation potential.

While cultural and societal readiness were identified as critical barriers, these were based on qualitative data from stakeholder perceptions. To further validate these dimensions, quantitative methods such as surveys or behavioral data would be needed to systematically assess public trust and cultural attitudes across sectors.

The framework was validated within a European policy context, influenced by regulations like the Data Governance Act. Its applicability in non-EU or less regulated contexts is unclear, and further research is needed to explore how the framework applies to diverse regulatory and institutional settings.

Despite these limitations, the SSH Framework offers a valuable tool for understanding the non-monetary drivers of data sharing. Future research should focus on quantitative validation, testing the framework in diverse contexts, and exploring how policy tools can bridge the gap between research findings and real-world implementation.

## 6. Discussion: Key insights from the SSH framework for data sharing

This chapter discusses the implications of applying the SSH Framework across six sectoral pilots, integrating evidence from framework validation, future visioning, and feasibility assessment. The analysis also highlights which dimensions of data sharing are currently most mature, which remain challenging, and how the framework contributes to understanding data sharing as a socio-technical and institutional practice rather than a purely technical one.

Across sectors, Ethics, Policy, and Scientific–Research emerged as the most consistently feasible dimensions, reflecting strong alignment between regulatory environments, institutional arrangements, and organisational priorities. Macro-economic aspects were also generally viewed as enabling rather than constraining. By contrast, Cultural and Societal dimensions systematically received lower feasibility ratings, indicating persistent barriers tied to trust, cultural norms, and legitimacy, rather than to infrastructure or regulation. Taken together, the findings suggest that while formal and technical foundations for data sharing are comparatively mature, the societal conditions for large-scale and sustained adoption remain uneven.

The feasibility patterns indicate that readiness is shaped by both structural conditions (e.g., governance maturity, regulatory alignment, organisational capacity) and motivational factors (e.g., trust, perceived fairness, expected benefit). This interplay is essential for advancing data governance approaches that are not only operationally effective but also socially legitimate and resilient.

### 6.1 RQ1 – Interpreting the key dimensions influencing data sharing

The empirical results confirm the findings from Zuiderwijk et al. 2020, ^20^ Devriendt et al. 2021, ^34^ and OECD 2022 ^35^ that data sharing is influenced by a constellation of societal, ethics, cultural, and organisational factors rather than by technical or economic considerations alone. The eleven SSH dimensions identified in this study provide a comprehensive framework for understanding these influences and align with scholarship that frames data sharing as a socio-technical and institutional phenomenon. ^3, 5^

**Trust, Transparency, and Ethics as Foundational Elements**

Across all pilots, trust, transparency, and ethical governance emerged as decisive factors shaping willingness to share data. In agriculture, for example, concerns about ownership, misuse, and unequal benefit distribution constrained engagement, underscoring that trust must precede participation rather than emerge as its outcome. These findings reinforce the view that ethical safeguards and transparent governance arrangements are essential for legitimacy, particularly in contexts marked by power asymmetries and information imbalance. ^7^ This mirrors the findings from Evertsz et al. (2023), ^36^ where trust and transparency were identified as critical enablers for data-sharing in public health research. Moreover, privacy protection and ethical governance are core to fostering institutional legitimacy in contexts such as healthcare and agriculture, where sensitive data is involved.

**Organisational Readiness as a Critical Enabler**

Organisational readiness like manifest in governance structures, defined responsibilities, internal processes, and institutional capacity, was consistently associated with implementation feasibility. The experience of HEDNO, where formal data-governance roles and automation structures were introduced, illustrates how institutional design can enable or inhibit data-sharing practices. This finding aligns with O’Reilly et al. (2020), who stress that institutional readiness plays a vital role in the effective deployment of data governance frameworks across sectors. ^37^

**Cultural Readiness and Societal Acceptance as Systemic Barriers**

Cultural and societal dimensions were consistently rated as less feasible than other dimensions. Resistance was driven not by technological constraints but by concerns regarding fairness, autonomy, accountability, and trust. In agriculture in particular, reluctance was linked to professional identity and perceived asymmetries in control and benefit distribution. Without deliberate interventions, such as data-literacy initiatives, participatory governance mechanisms, and transparent consent frameworks, cultural resistance is likely to persist even when supporting infrastructure and regulation are in place. This issue has been highlighted in recent studies, including Taylor and Broeders (2015), where cultural resistance and concerns about data ownership limited the effectiveness of data-sharing systems in global development contexts. ^38^

**Scientific–research, Ecological, and Macro-Economic Value**

Strong scientific–research and ecological value propositions were evident in meteorology and energy, while macro-economic benefits featured more prominently in agriculture and telecommunications. These patterns align with contemporary policy discourse positioning data as an enabler of societal and ecological value beyond economic growth alone as demonstrated by OECD (2022) in their recent policy paper on enhancing access to and sharing of data for sustainable governance. ^13, 24^ The framework’s capacity to capture diverse value logics across sectors supports its applicability in heterogeneous governance contexts, as discussed by Micheli et al. (2020) in the context of emerging models of data governance in data-driven sectors. ^33^

**Interdependencies Within the SSH Framework**

The findings confirm that the SSH dimensions constitute a connected system rather than discrete variables. Governance arrangements influence trust; ethical standards shape regulatory confidence; cultural values mediate public acceptance; economic benefits depend on customer confidence; and organisational maturity reflects regulatory clarity. Recognising these interdependencies supports a shift from isolated interventions toward integrated governance strategies, in line with Janssen et al. (2017), who advocate for integrated governance frameworks in open government to enhance trust and transparency. ^21^

### 6.2 RQ2 – Framework applicability for future visioning

The use of the SSH Framework in participatory foresight confirms its value as a tool for future-oriented strategic reflection.

**Translating SSH Concepts into Practice**

Across pilots, stakeholders actively employed the framework to articulate desirable data-sharing futures. The dimensions facilitated structured reflection on governance, ethics, culture, and public value, demonstrating that SSH concepts can be translated into actionable planning instruments. This strengthens the framework’s relevance for operational governance processes. This highlights the importance of embedding SSH concepts in data governance models to support meaningful policy development and operationalisation, as discussed by Micheli et al. (2020). ^33^

**Diversity and Convergence Across Sectors**

Although future visions reflected sector-specific priorities and recommendations, ranging from competitiveness and innovation to public interest and ecological sustainability, a consistent set of recommendations recurred:

• scalable and secure infrastructure
• data quality, interoperability, and accessibility
• transparent and ethical governance
• cross-sector collaboration
• organisational capacity building
• alignment with EU regulatory frameworks

This convergence suggests the emergence of a shared understanding of key conditions for trusted data ecosystems, while remaining differences confirm the framework’s adaptability. These patterns of convergence across sectors align with the research by Zuiderwijk et al. (2020), which found that data sharing across sectors requires adaptable frameworks that reflect both commonalities and unique sectoral needs. ^20^

**A Common Baseline for Sector-Specific Futures**

The framework enabled pilots to develop coherent yet differentiated visions. This flexibility is particularly valuable in the context of European data spaces, which require sector-specific architectures under shared governance principles. The SSH framework’s flexibility aligns with work by Janssen et al. (2017), which argued that successful European data governance must accommodate sector-specific needs while maintaining an overarching regulatory and operational coherence. ^21^ The framework thus contributes to anticipatory governance by supporting systematic reflection on societal and institutional dimensions alongside technical planning.

### 6.3 RQ3 – Feasibility patterns and readiness for implementation

The feasibility assessment highlights uneven readiness across SSH dimensions.

**High-Feasibility Dimensions**

Ethics, policy, and scientific–research consistently received the highest feasibility ratings. These dimensions benefit from institutional anchoring, regulatory momentum, and alignment with organisational mandates, positioning them as effective entry points for near-term implementation. The high feasibility of these dimensions resonates with the findings of Devriendt et al. (2021), where ethical and policy dimensions were similarly identified as foundational for facilitating data-sharing in research contexts. ^34^

**Moderate-Feasibility Dimensions**

Macro-economic, organisational, and customer–business dimensions varied across sectors. Higher readiness in energy and telecommunications contrasted with challenges in agriculture and infrastructure, illustrating differentiated sectoral capacity and the need for bespoke support mechanisms. This aligns with the observations of Popper (2008), who highlights that sectoral maturity and institutional capacity significantly affect the feasibility of implementing cross-sector governance initiatives. ^15^

**Low-Feasibility Dimensions: Societal and Cultural Readiness**

Cultural and societal dimensions were persistently rated as least feasible. Barriers were primarily institutional and normative rather than technical. Public trust, fairness perceptions, and cultural norms shaped adoption expectations. Addressing these constraints therefore calls for sustained engagement strategies, societal value demonstration, and participatory governance rather than technology deployment alone. These findings align with Evertsz et al. (2023), who identify cultural resistance and trust deficits as significant barriers in the effective adoption of data-sharing frameworks in public health research. ^36^

Overall, feasibility patterns reaffirm that implementation readiness emerges from the interaction between formal governance conditions and lived experience, reinforcing the need for integrated policy design. This resonates with the conclusions of Janssen et al. (2017), who emphasized the importance of aligning technical, social, and institutional dimensions for effective implementation. ^21^

### 6.4 Implications for data governance and EU policy alignment

The feasibility patterns identified across sectors reveal strong alignment between the SSH Framework and the EU data governance landscape. High feasibility in Ethics, Policy, and Scientific–Research reflects convergence with the principles embedded in the Data Governance Act, the Open Data Directive, and the European Strategy for Data. ^1, 2^ Ethical governance practices identified in the pilots - such as transparency mechanisms, accountability structures, and fairness-oriented data management - directly reflect EU objectives of building trustworthy and human-centric data ecosystems. This alignment with EU policy frameworks is also underscored by the OECD (2022), which advocates for the adoption of transparent governance practices and the integration of ethical principles in data sharing. ^11^

Strong readiness in the Policy and Scientific–Research dimensions further illustrates the enabling role of regulatory clarity and institutional mandates. Public-sector and research-driven pilots benefit from alignment with European data spaces, FAIR data principles, and open science frameworks. EU regulation thus operates not merely as a compliance mechanism, but as an active governance infrastructure shaping coordination and interoperability as shown by Micheli et al. (2020) in their analysis of emerging governance models for open data. ^33^

By contrast, Cultural and Societal dimensions remain the least feasible across sectors. Barriers in these areas reflect deficits in trust, legitimacy, and perceived fairness rather than technical limitations. In agriculture in particular, concerns related to data ownership, value distribution, and autonomy limit participation despite regulatory and technical progress. These findings indicate that compliance-based governance alone is insufficient where societal legitimacy is weak. This finding is consistent with the observations made by Taylor and Broeders (2015), who note that cultural legitimacy is a critical factor that must be addressed alongside technical solutions. ^38^

Strengthening cultural and societal readiness therefore requires complementary governance strategies. EU-level instruments could include data literacy initiatives, participatory governance models within European data spaces, guidance on ethical-by-design practices, and societal impact assessment in data infrastructure development. These strategies align with the broader EU ambition of embedding responsibility and inclusion into digital transformation. ^17^

Overall, while the institutional foundations of European data spaces are increasingly robust, long-term sustainability depends on addressing cultural legitimacy and organisational readiness alongside regulatory harmonisation. The SSH Framework helps identify where regulation is effective and where sociocultural capacity building is still required.

**6.4.1 Acknowledging limitations in empirical base and scope of claims**

This study provides valuable insights into the dimensions shaping data sharing across six pilot sectors. However, as noted, the small-N qualitative sample and reliance on self-reported feasibility scores impose important limitations on the broader applicability of these findings. The findings are based on a specific set of pilot sectors (energy, telecommunications, agriculture, infrastructure, and meteorology), and while these sectors were chosen for their involvement in the DATAMITE project, they may not fully represent other sectors or data-sharing practices within the EU or beyond. Consequently, the conclusions drawn from this study are context-specific and should not be overly generalised to all potential data-sharing ecosystems.

Moreover, the self-reported feasibility scores, which reflect perceived readiness for implementation rather than actual outcomes, should be interpreted with caution. These scores may be subject to optimism bias, sectoral interests, or differing interpretations of what constitutes “feasibility,” and therefore do not provide definitive measures of actual implementation. The feasibility ratings represent perceptions of readiness and should be seen as indicative rather than conclusive. Such self-reported biases are well-documented in data governance studies, including those by Popper (2008), who cautioned that subjective assessments should be interpreted with care in strategic foresight contexts. ^15^

Given these limitations, the conclusions of this study must be viewed as context-specific and not overly generalized. The empirical evidence here, while valuable, is based on a small sample of six pilots in specific sectors, and broad generalisations across sectors or countries should be avoided without further research. Future studies with larger samples, perhaps incorporating quantitative data or longitudinal case studies, are needed to validate and extend these findings and to test the framework’s applicability in other sectors and regions.

### 6.5 Overall synthesis

The SSH Framework offers a robust analytical lens for conceptualising data sharing as a socio-institutional system rather than a technical pipeline. It enables both diagnostic assessment and strategic design by linking empirical insight with future-oriented governance. By making societal sustainability explicit in data governance, the framework contributes to the European ambition of building federated, trustworthy, and human-centric data spaces.

## 7. Conclusion

This study developed and validated a Social Sciences and Humanities (SSH) Framework for Data Sharing that captures the non-monetary conditions underpinning trusted and sustainable data ecosystems. By distinguishing eleven SSH dimensions, the framework extends existing approaches beyond technical and economic perspectives to account for societal, ethics, cultural, and organisational influences on data-sharing practices.

Empirical validation across six European pilots confirmed that all dimensions are observable in practice, while demonstrating substantial variation in readiness across five domains. The framework’s application through participatory foresight further showed that SSH dimensions are not only analytically meaningful but operationally useful for developing sector-specific futures and assessing implementation feasibility. This aligns with the perspectives outlined by Zuiderwijk et al. (2020), who stressed the importance of integrating societal and cultural factors into data governance to promote long-term sustainability in data sharing. ^20^

In that context, the framework may complement and expand existing approaches such as identity and trust infrastructures (e.g., DID/VC ecosystems ensuring decentralized identity ^39^ maturity models ^40^ compliance-by-design ^41^ or privacy-by-approaches in the design phase of business processes and technical development. While these approaches assess performance in a structured way and tackle issues of security, legal compliance and privacy on a formal level, our SSH framework aims at considering broader impacts in a systematic way for strategic scoping. This mirrors the recommendations from Janssen et al. (2017), who highlighted the need for governance models that integrate both technical and socio-cultural dimensions to ensure data sharing practices remain effective and inclusive in the long run. ^21^

From a governance perspective, the framework identifies where EU data policy is already supported by institutional readiness, particularly in ethics, policy alignment, and scientific–research, and where persistent gaps remain, notably in cultural and societal readiness. These findings support the OECD’s (2022) conclusion that while technical frameworks are well-established, the most significant challenges for sustainable data governance lie in addressing societal and cultural barriers, such as trust and legitimacy. ^11^ These results underscore that regulatory harmonisation and technical interoperability alone are insufficient for achieving sustainable data sharing without corresponding investment in trust, participation, and organisational capacity.

Thus, the SSH Framework offers a practical tool for assessing sectoral readiness and prioritising sociocultural capacity-building alongside regulatory development. It supports differentiated governance strategies rather than uniform solutions, recognising that sectoral diversity requires flexible and context-sensitive policy design. As seen in the work of Evertsz et al. (2023), flexible governance strategies are crucial for addressing sector-specific challenges and fostering engagement in data-sharing initiatives, particularly in complex, multi-stakeholder environments. ^36^

Future research should extend validation beyond the DATAMITE context and develop quantitative indicators for measuring SSH dimensions more systematically. Comparative studies across regulatory environments would further strengthen the framework’s generalisability and enable assessment of how sociocultural conditions shape implementation under different governance regimes.

## Ethics and consent

The study involved discussions with relevant stakeholders. No human participants or personal data were included. All stakeholder input was provided voluntarily, and informed consent was obtained where applicable. Ethical approval was not required for this study.

The study adhered to the institutional ethical guidelines, and a consent form was provided in accordance with these standards. All participants taking part in the interviews provided written informed consent prior to interviews and workshops. The consent process included a full briefing on the purpose of the research, the use of their contributions, and the measures in place to protect their privacy. Consent was obtained with the clear understanding that all data would be anonymized. Participants were informed that they could withdraw their data at any stage by submitting a written request to the corresponding author. Personal identifiers were anonymized with the Safe Harbor method.

## AI and generative AI use declaration

During the preparation of this manuscript, the authors used ChatGPT (version 4.0) for language editing and proofreading purposes. The AI tool was employed to review and refine the English language expression, grammar, and clarity of the written text while preserving the original meaning and scientific content. All substantive research content, analysis, interpretation of results, and conclusions presented in this work are entirely the authors’ own. The use of this generative AI tool was limited to language enhancement and did not contribute to the conceptual framework, data collection, analysis, or interpretation of findings.

## Data Availability

This study is based on qualitative data collected within Work Package 4 of the DATAMITE project, covering six pilots as described. The dataset consists of list of identified literature, anonymized semi-structured interviews for visions and workshop outputs. To protect participant confidentiality, all interview data were fully anonymized before analysis. Personal identifiers were removed or replaced with codes, and any context that might allow indirect identification was carefully reviewed. Metadata describing the dataset follows FAIR principles, ensuring that it can be discovered and reused under appropriate conditions. The datasets generated and analysed during the current study are freely available under a CC-BY 4.0 International License. All data are publicly accessible, and the datasets can be accessed via the following persistent identifiers or through the Zenodo repository. The data are open for reuse, redistribution, and modification, provided appropriate attribution is given, in line with the conditions of the CC-BY 4.0 licence. For additional requests or specific data subsets not available in the repository, please contact the research coordinator, Margit Hofer ( hofer@zsi.at), at the Centre for Social Innovation (ZSI). This study is based on a review of literature and qualitative data collected within Work Package 4 of the DATAMITE project, covering six pilots as described. The dataset consists of a) visions described by the pilots and workshop outputs from quantified recommendations b) a set of identified literature c) a set of pictures and graphs. The data can be accessed via the following persistent identifiers or through the Zenodo repository under Centre for Social Innovation (2025): The data can be accessed via the following persistent identifiers or through the Zenodo repository: This project contains the following underlying data:
a) Data Set on Visions and Recommendations incl. Focus group working template and consent file template: https://doi.org/10.5281/zenodo.19367027 ^42^
b) Literature Data set: https://doi.org/10.5281/zenodo.19255233 ^43^
c) Pictures/Graphs used for this publication: https://doi.org/10.5281/zenodo.19248216 ^44^ Data are available under the terms of the Creative Commons Attribution 4.0 International license (CC-BY 4.0).
