# Supplementary material for: Mapping Non-Monetary Values in Data Sharing: Development and Validation of an SSH Framework Across Sectors
Source: Open Res Eur. 2026 May 26;6:177. [Version 1] doi: 10.12688/openreseurope.23548.1 (PMC13550886; doi:10.12688/openreseurope.23548.1)
Supplement: Peer review history

# Reviewer report: Reviewer response for version 1

**DOI:** 10.21956/openreseurope.25494.r75137

**Published:** 2026-07-10

**Authors:** Sarah-Louise Ruder

**Affiliations:** 1 University of Ottawa, Ottawa, Ontario, Canada; 2 Sociology, University of Ottawa, Ottawa, Ontario, Canada

Thank you for the opportunity to review your article “Mapping Non-Monetary Values in Data Sharing: Development and Validation of an SSH Framework Across Sectors.” The article is well written and offers a useful cross-sector analysis on data sharing. I have several suggestions to clarify and strengthen the message.

 Greater specificity needed:

- The article could benefit from greater clarity in terms for a broad audience, e.g. “data governance” is used without a clear definition. What are the monetary values in data sharing? If you are emphasizing non-monetary values, I think the article would benefit from a short summary of existing frameworks with monetary values. While the focus on non-monetary values is justified and needed, data monetization (as well as commodification and ‘assetization’) is still an open question in many sectors.
- A definition of “values” could also be helpful in section 1 or 2. Why did you choose values? There are many other non-monetary aspects of data sharing and data governance.
- Does it matter what specific data types, formats, resolution, etc. are at hand in each sector? Some sectors are more likely than others to handle personal or biometric data with different legal framework for its use, versus non-personal data.
- It would be helpful to an international audience to have a bit more context on DATAMITE and the pilots.

3. Methods:

- It is a matter of style, but I recommend re-ordering the content in section 3. It would be more streamlined to present the design, data collection, and analysis all at once for each component of the methods chronologically (e.g., everything about the literature review, then the interviews and questionnaires, then workshops).
- The search terms “macroeconomic” and “societal” may have been more limiting than “economic” (not limited in scale) and “soci*” (including social). If you agree, this could be mentioned as a limitation.
- Table 1 might be missing a database. There is an empty cell in the left most column.
- It was not immediately clear to me if the interviews and survey were completed by the same people; I suggest saying it explicitly.
- How many workshops did you complete? Who participated? Was it all 46 DATAMITE partners? How were they recruited and selected?
- It is good practice to have the full list of included sources for a systematic review as available supplemental material.

5. Results

- Did you collect qualitative data during the interviews and workshops to supplement the results section? It would be great to have a few quotations or summarized examples from the pilots to further characterize your SSH Framework. For example, you have the three bullet points with synthesis across the six pilots, but how do these come up in practice? What were the similarities and differences across sectors/pilots?

5. Limitations:

- I recommend either moving section 5 into the discussion with what is now 6.4.1. “Acknowledging limitations in empirical base and scope of claims” or moving 6.4.1 into section 5. At present it is distracting to have limitations in two different sections.
- I think the sample size should be mentioned in section 5. While the sample size is limited, the combination of methods is effective. I still think the authors should comment on how confident that a single pilot per sector (and 1-2 people per pilot) can support conclusions that compare sectors.
- It is also good practice to acknowledge the language limitations in systematic reviews as potentially excluding relevant research in other languages.

Overall, the Discussion and Conclusion work well. I did wonder whether the “social science and humanities” label is most appropriate your framework. The engagement with social science and humanities literature seems mostly about selecting key topics, rather than engaging with the theory and empirical SSH work to frame the issues around data sharing. I noticed that much of the works references are from Europe (which makes sense and is likely best for this outlet), but it could be worth connecting to other regions for your Discussion. My relevant expertise for this review is on agriculture data governance. I could see many connections to the work in this space from North America, namely Kelly Bronson, Michael Carolan, Maywa Montenegro de Wit, and from Australia, namely Leanne Wiseman and Emma Jakku. Of course there is an entire field of Critical Data Studies, not limited to food and agriculture, that would also be relevant here.

 Figures

- Figure 1: Resolution for the image is not sufficiently clear. The inconsistent capitalization is distracting. The left side seems too tightly cropped and cuts off part of the words.
- Figure 2: The other of the numbered boxes is unclear/confusing. It would be better to have the visual aligned with the five SSH dimensions listed in 4.1.2.1. Then you could have labels for the items that fit within each category.
- Figure 4: The numbers are quite difficult to read. The resolution for the image is not suitable for indexing.

If applicable, is the statistical analysis and its interpretation appropriate?

Not applicable

Is the study design appropriate and is the work technically sound?

Yes

Is the work clearly and accurately presented and does it engage with the current literature?

Partly

Are the conclusions drawn adequately supported by the results?

Yes

Are sufficient details of methods and analysis provided to allow replication by others?

Partly

Are all the source data and materials underlying the results available?

No

Reviewer Expertise:

agricultural data governance; data sharing; critical data studies; qualitative and quantitative social science methods

I confirm that I have read this submission and believe that I have an appropriate level of expertise to confirm that it is of an acceptable scientific standard, however I have significant reservations, as outlined above.

# Reviewer report: Reviewer response for version 1

**DOI:** 10.21956/openreseurope.25494.r75133

**Published:** 2026-06-16

**Authors:** Andrea Farina

**Affiliations:** 1 King's College London, London, England, UK

The paper develops and validates an 11-dimension Social Sciences and Humanities (SSH) Framework for Data Sharing, grounded in a systematic literature review, stakeholder interviews across six European pilots, and participatory foresight. It addresses three research questions about which SSH dimensions influence data sharing, whether the framework generates useful sector-specific visions, and which dimensions pose the greatest implementation challenges.

 The authors can find some points for suggestions below. As I didn't have any .docx/.pdf of the manuscript, I'll mention sections and quote parts of the manuscript before writing my comments.

 Section 3

- "A systematic and transparent literature review was conducted as part of the DATAMITE foresight process. Its purpose was to identify non-monetary impacts of data sharing, explore sector-specific patterns, and establish a conceptual foundation for subsequent empirical inquiry”: **what type of data sharing are we talking about? Are we looking at data shared within repositories, data papers, white papers, etc.?**
- "Coding reliability was ensured through inter-coder agreement”: **using which metrics and what was the mathematical result yielded by these metrics?**

- "The search strategy was clearly defined using the PICOC methodology28,29 (Population, Intervention, Comparison, Outcomes, Context), ensuring the inclusion of studies with rigorous methodological approaches and relevance to the research questions.”: **The PICOC methodology could be briefly described to clarify this section.**
- In general, I believe all these subsections in 3.1 break the narrative and make the section more difficult to understand. I’d make the whole section slightly more narrative.
- "Out of these 253, 40 studies were selected by a core team based on title, abstract, and keywords, using criteria of methodological rigor and relevance to the research question”: I believe the choice of 40 studies out of 253 should be better addressed: **why 40? what are the "criteria of methodological rigor” mentioned by the authors.**
- "Finally, each pilot self-assessed the feasibility (i.e., perceived ability of implementation, not actual implementation) of its recommendations using a six-point Likert scale (1 = very low, 6 = very high)”. **How do the authors account for potential biases (such as optimism biases) given that this is self-assessed? I see the authors mention this later in Section 5, but the analytical implications are underplayed.**

Section 4

- "The thematic synthesis of 40 peer-reviewed studies yielded an initial set of eight non-monetary dimensions, later expanded to eleven: societal, ecological, security, scientific–research, macro-economic, customer–business, policy, ethics, cultural, organizational, and regulatory. “: **why were these eight non-monetary dimensions expanded? were these 8/11 assumed or derived? what do these dimensions actually include? a clearer theoretical rationale for where one dimension ends and another begins would strengthen the framework's analytical precision. Also, my concern is that the 11 dimensions found by the authors read more like a social science inventory than an SSH one… Anything distinctively humanities in character is absent from the list (e.g., history, philosophy, literature, languages etc.). Perhaps the authors would like to elaborate on their terminological choice or just use “Social Science”?**
- Figure 4: please enhance figure quality.

Section 5

- I’d suggest the authors move the section at the end of the paper.

Section 6

- "Ethics, policy, and scientific research consistently received the highest feasibility ratings.”: see my point on boundary definition of dimensions in Section 4. Ethics and policy seem two very closely related categories, so this might be the reason for receiving similar scores (in other words, ethics may score well *because of* policy infrastructure). Better discussing dimension boundaries would help the reader understand the final discussion as well.
- In general, I believe all these subsections in 6.1-6.3 break the narrative and make the section more difficult to understand. My suggestion here is to better tight the findings to the RQs in such a narrative way that the reader could in fact grasp the paper findings, which are currently too much broken down, in my opinion.
- **Section 6.4.1 seems like a repetition of Section 5**.

General suggestions:

- The paper is quite long and repetitive e.g., when it comes to restating the same core argument in different sections (or see my comment above for sections 6.4.1 and 5). Tightening the prose would sharpen the contribution considerably and make the authors’ claim easier to grasp (this is also linked to my suggestions above about rephrasing some sections to make them more narrative).
- The authors note in the data availability statement that the dataset used for the study is currently available. It would be great to include the dataset reference within the text too, maybe in Section 3. In case the authors have already quoted is as a reference, I might have missed it due to quite inconvenient format by which references are quoted in the paper!

If applicable, is the statistical analysis and its interpretation appropriate?

Not applicable

Is the study design appropriate and is the work technically sound?

Partly

Is the work clearly and accurately presented and does it engage with the current literature?

Yes

Are the conclusions drawn adequately supported by the results?

Partly

Are sufficient details of methods and analysis provided to allow replication by others?

Partly

Are all the source data and materials underlying the results available?

Yes

Reviewer Expertise:

Computational Linguistics, Open Data / Open Science

I confirm that I have read this submission and believe that I have an appropriate level of expertise to confirm that it is of an acceptable scientific standard, however I have significant reservations, as outlined above.

# Reviewer report: Reviewer response for version 1

**DOI:** 10.21956/openreseurope.25494.r75134

**Published:** 2026-06-08

**Authors:** Anna Essén

**Affiliations:** 1 Stockholm School of Economics, Stockholm, Sweden

Thank you for providing me with the opportunity to read this study! A key strength of this study is its contribution to the data governance literature by shifting attention from predominantly technical, legal, and economic concerns toward the non-monetary dimensions that shape the success of data sharing initiatives. While existing data governance frameworks often emphasize interoperability, compliance, access rights, and value creation, this study demonstrates that governance effectiveness also depends on factors such as ethics, trust, organisational capacity, cultural readiness, and societal legitimacy. By developing and empirically validating an SSH framework across six sectors, the study provides a more holistic understanding of governance as a socio-technical process. The identification of eleven interconnected dimensions offers a valuable analytical lens for examining how data-sharing arrangements become accepted, sustained, and scaled in practice, thereby extending current discussions on trustworthy and human-centric data governance.

 The study also makes an important contribution to the broader data sharing literature by addressing the persistent challenge of explaining why technically feasible data-sharing initiatives often struggle to achieve long-term adoption and impact.

 Through its combination of literature review, stakeholder interviews, and participatory foresight methods, the study highlights how social, organisational, and institutional conditions influence willingness and capacity to share data. The cross-sectoral validation strengthens the framework's relevance beyond a single domain and demonstrates the importance of considering governance readiness, trust relations, and cultural factors alongside technical infrastructures.

 In doing so, the study advances emerging scholarship that conceptualises data sharing as a socio-technical and institutional practice rather than merely a problem of technological interoperability.

 The methodology is generally well described and benefits from a transparent multi-method design combining literature review, interviews, stakeholder feedback, and participatory foresight activities. However, the paper could further strengthen methodological transparency by providing additional detail on the selection of interview participants, the criteria used to choose the final 40 articles included in the literature review, and the procedures used to ensure consistency in coding and interpretation across researchers. Could be in an appendix. Sorry if I misunderstood/missed this.

 Greater information about the composition of workshop participants, the feasibility assessment process, and how disagreements were resolved during coding and validation would also enhance the study's reproducibility. While the iterative framework development is a notable strength, a more explicit account of how insights from each methodological stage informed subsequent revisions of the SSH framework would make the analytical process easier for readers to follow and evaluate.

 One issue with the paper is that it is a bit generic. One way of addressing this could be to use an empirical case to illustrate several of the challenges? The same case could be use (fictive or real) to illustrate the points made. I do however understand that space is limited. Perhaps for the most pressing challenges, to make them come alive more and show their urgency.

 To further anchor the paper in the data governance literature in IS and data studies, see suggested review below. The work of Jarvenpaa et al could provide a valuable complement to the current framework. For instance, their work (see refs below) argues that data governance research tends to overlook temporal complexity and often assumes a single, present-oriented conception of time. It introduces the concepts of temporal complexity and temporal fragility, showing how data-sharing arrangements must accommodate multiple temporal orientations, rhythms, and horizons among stakeholders.

 This perspective could enrich the discussion of organisational, cultural, governance, and sustainability dimensions by highlighting how data-sharing infrastructures must remain useful and legitimate not only across organisations but also across time.

 Relatedly, work on data sustainability offers an important extension of the paper's concern with socially sustainable data spaces. Rather than focusing solely on current sharing arrangements, data sustainability emphasises the continuous work required to maintain data accessibility, usability, and relevance for future stakeholders.

 This perspective could strengthen the paper's treatment of long-term governance by connecting organisational readiness and governance capacity to the ongoing maintenance, curation, and preservation efforts necessary to ensure that data remain valuable across changing technological, organisational, and societal contexts.

 Suggested some Refs that may be of interest Ref 1, 2, 3 and 4. 

If applicable, is the statistical analysis and its interpretation appropriate?

Not applicable

Is the study design appropriate and is the work technically sound?

Yes

Is the work clearly and accurately presented and does it engage with the current literature?

Partly

Are the conclusions drawn adequately supported by the results?

Partly

Are sufficient details of methods and analysis provided to allow replication by others?

Yes

Are all the source data and materials underlying the results available?

Partly

Reviewer Expertise:

NA

I confirm that I have read this submission and believe that I have an appropriate level of expertise to confirm that it is of an acceptable scientific standard.

## References

[1] : A Systematic Review of Recent Literature on Data Governance (2017–2023). *IEEE Access* .2024;12: 10.1109/ACCESS.2024.3476373 149875-149888 10.1109/ACCESS.2024.3476373

[2] : Data Governance as a Collective Action Problem. *Information Systems Frontiers* .2020;22(2) : 10.1007/s10796-019-09923-z 299-313 10.1007/s10796-019-09923-z

[3] : Data sustainability: Data governance in data infrastructures across technological and human generations. *Information and Organization* .2023;33(1) : 10.1016/j.infoandorg.2023.100449 10.1016/j.infoandorg.2023.100449

[4] : Temporal fragilities in data sharing and data governance.2026; 10.4337/9781035348718.00025 218-232 10.4337/9781035348718.00025
